# Differential Regulation of Starch-synthetic Gene Expression in Endosperm Between *Indica* and *Japonica* Rice Cultivars

**DOI:** 10.1186/s12284-017-0146-5

**Published:** 2017-02-28

**Authors:** Tsuyoshi Inukai

**Affiliations:** Research Faculty of Agriculture, Hokkaido University, Sapporo, 060-8589 Japan

**Keywords:** Starch accumulation rate, Amylose, Amylopectin, Sucrose, Sugar signal

## Abstract

**Background:**

Grain filling rates (GFRs) of *indica* rice cultivars are often higher than those of *japonica* cultivars. Although GFR is mainly determined by the starch accumulation rate (SAR) in endosperm, the genetic basis for SAR during the ripening period has not been well studied in rice. To elucidate the factors influencing the differing SARs between typical *indica* and *japonica* cultivars, we focused on differences in sink potentials, especially on starch synthesis in the endosperm.

**Results:**

SAR in *indica* rice cultivar IR36 was significantly higher than in *japonica* cultivar T65. Although enzymes for both amylose and amylopectin syntheses had higher activity in IR36, amylopectin synthesis was seemingly more important for accelerating SAR because an elevation of amylose synthesis ability alone in the T65 genetic background did not result in the same level of SAR as IR36. In IR36, most starch-synthetic genes (SSGs) in the endosperm were more highly expressed during ripening than in T65. In panicle culture experiments, the SSGs in rice endosperm were regulated in either sucrose-dependent or -independent manners, or both. All SSGs except *SSI* and *BEIIa* were responsive to sucrose in both cultivars, and *GBSSI, AGPS2b* and *PUL* were more responsive to sucrose in IR36. Interestingly, the *GBSSI* gene (*Wx* ^*a*^) in IR36 was highly activated by sucrose, but the *GBSSI* gene (*Wx* ^*b*^) in T65 was insensitive. In sucrose-independent regulation, *AGPL2*, *SSIIIa*, *BEI*, *BEIIb* and *ISA1* genes in IR36 were upregulated 1.5 to 2 times more than those in T65. Additionally, at least *SSI* and *BEIIa* might be regulated by unknown signals; that regulation pathway should be more activated in IR36 than T65.

**Conclusions:**

In this study, at least three regulatory pathways seem to be involved in SSG expression in rice endosperm, and all pathways were more active in IR36. One of the factors leading to the high SAR of IR36 seemed to be an increase in the sink potential.

**Electronic supplementary material:**

The online version of this article (doi:10.1186/s12284-017-0146-5) contains supplementary material, which is available to authorized users.

## Background

Starch in rice endosperm is synthesized via the coordinated activities of several enzymes (Jeon et al. 2010). ADP-glucose, serving as the glucose donor for starch synthesis, is mainly synthesized in cytoplasm by the ADP-glucose pyrophosphorylase (AGPase), that is a heterotetramer consisting of two small subunits (AGPS2b) and two large subunits (AGPL2). After ADP-glucose is transported from the cytoplasm to amyloplasts, glucan chains with a certain degree of polymerization are first synthesized as primers for starch synthesis by plastidial starch phosphorylase (Pho1) (Satoh et al. 2008). Starch consists of two types of glucan polymers: amylose and amylopectin. Amylose comprises predominantly linear chains of α(1–4)-linked glucose residues and is synthesized by granule-binding starch synthase I (GBSSI) encoded by the *Wx* gene (Jeon et al. 2010). While the wild type allele *Wx* ^*a*^ is found in most rice cultivars belonging to *indica* subspecies, the mutant allele *Wx* ^*b*^ is widely distributed in *japonica* subspecies (Sano 1984; Sano et al. 1991). *Wx* ^*b*^ possesses a G to T mutation at the 5′ splicing site of the first intron, which leads to a decrease in the splicing efficiency (Bligh et al. 1998; Cai et al. 1998; Isshiki et al. 1998; Hirano et al. 1998). Therefore, the GBSSI activity of *japonica* is considerably weak and results in starch with a low amylose content. Amylopectin has a multiple cluster structure consisting of a highly branched glucan with α-1,6-glucosidic bonds (Jeon et al. 2010), and its synthesis is coordinately catalyzed by three classes of enzymes: soluble starch synthases (SSs: SSI, SSIIa and SSIIIa), starch branching enzymes (BEs: BEI, BEIIa, BEIIb) and starch debranching enzymes (isomerase 1 [ISA1] and pullulanase [PUL]) (Jeon et al. 2010). While SSs catalyze the elongation reaction of α(1–4)-linked glucose residues, BEs introduce α-1,6-glucosidic bond to them. ISA1 and PUL remove unnecessary α-1,6-glucosidic bonds that interfere with formation of normal amylopectin clusters. For SSIIa, four amino acid (AA) substitutions exist between the *indica* and *japonica* cultivars (Nakamura et al. 2005); two of these substitutions are in the C-terminal region and are crucial for the SSIIa activity. Thus, the *japonica* cultivars lost almost all SSIIa activity, resulting in significant differences in the short to medium chain ratio within amylopectin clusters (Nakamura et al. 2005).

Important transcription factors that regulate the starch-synthetic genes (SSGs) have been identified in rice. RSR1 is a negative regulator of the SSGs in the endosperm, and the expression of all SSGs was upregulated in mutant *rsr1*, resulting in larger grains and higher grain weight and amylose content (Fu and Xue 2010). Alkaline leucine zipper transcription factor OsbZIP58 directly regulates *AGPL3*, *Wx*, *SSIIa*, *BEI*, *BEIIb* and *ISA2* in a positive manner (Wang et al. 2013). In *osbZIP58* mutants, starch and amylose contents were significantly lower than in the wild type. On the other hand, some SSGs such as the *Wx* and *BEIIb* genes are temperature-responsive (Hirano and Sano 1998; Yamakawa et al. 2007), and the promoter of the *Wx* ^*b*^ gene is responsive to cool temperatures (Hirano and Sano 1998). For transcriptional regulation of *Wx* ^*b*^, loci *du-1* and *du-2* might be involved as splicing factors in alternative splicing of pre-mRNA of *Wx* ^*b*^ (Isshiki et al. 2000).

The signaling pathway controlling starch synthesis in rice endosperm remains unclear. The expression level of plastidial *AGPL3* is synergistically regulated by both sucrose and abscisic acid (ABA) in cultured cells of rice (Akihiro et al. 2005). Recently, *ZmSSIIIa*, a maize homolog of the rice *SSIIIa*, was shown to be positively modulated by the ZmEREB156 transcription factor with synergistic regulation by sucrose and ABA (Huang et al. 2016).

Early maturation is one of the most important traits in rice breeding, especially in temperate regions where the optimum season for rice cultivation is often limited. Because the main target in breeding for maturity is the time required for heading, many genes for heading time have been cloned and the regulatory networks clarified (Tsuji et al. 2013; Matsubara et al. 2014; Shrestha et al. 2014). Although the grain-filling rate (GFR) during ripening also affects maturity, GFR is rarely a breeding target because the genetic basis for GFR is not well understood. In *indica*, genetic variation in GFR has often been reported to be higher than in *japonica* (Nagato and Chaudhry 1969; Yoshida and Hara 1977; Osada et al. 1983). Multiple factors such as photosynthesis activity in source organs, efficiency of sugar translocation and/or starch synthesis activity in sink organs appear to be involved in the difference in GFR between the two subspecies. However, Murchie et al. (2002) reported that the differences of GFR among rice cultivars is not explained by differences in source properties such as light-saturated rate of photosynthesis or in the level of ribulose 1,5 bisphosphate carboxylase oxygenase or total chlorophyll. Because differences in the sink potential, especially in the ability to synthesize starch, among rice cultivars have not been thoroughly studied, here we compared sink potentials between typical *indica* and *japonica* rice cultivars in terms of SSG regulation and found that the SSGs in endosperm were differentially regulated between the two rice cultivars.

## Results

### Differential Regulation of Starch-synthetic Gene Expression Between IR36 and Taichung 65 (T65)

In this study to compare indicators of starch accumulation in karyopses between *indica* and *japonica* rice cultivars under the same environmental conditions, indica cultivar IR36 and japonica cultivar T65 were selected because their vegetative growth in a greenhouse at Hokkaido University in Sapporo during the summer differed by only several days. To sample the spikelets flowering at the same time, sowing dates of those cultivars were adjusted. The mean temperature in the greenhouse during the summer was always over 25 °C, suitable for tropical cultivars IR36 and T65.

**Fig. 1:**
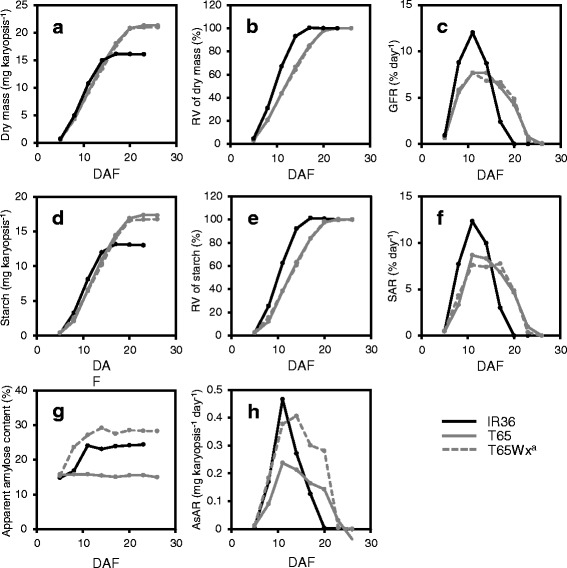
Starch accumulation in rice endosperm of *indica* cultivar IR36, *japonica* cultivar T65 and T65 near-isogenic line T65Wx^a^ over time during ripening. **a** Dry mass. **b** Relative value (RV) of dry mass. **c** Grain filling rate (GFR) calculated from data in panel **b**. **d** Starch content. **e** RV of starch content. **f** Starch accumulation rate (SAR) calculated from data in panel **e**. **g** Apparent amylose content. **h** Amylose accumulation rate (AsAR) calculated from data in panels **d** and **g**. DAF, days after flowering

Both the dry mass of the karyopsis and the amount of starch in the endosperm of IR36 peaked (16.1 and 13.0 mg karyopsis^−1^) about 1 week earlier than in T65 (21.3 and 17.3 mg karyopsis^−1^) (Fig. 1a and d). The difference in grain weight between IR36 and T65 was relatively large, suggesting that the number of endosperm cells also differed significantly between the two cultivars because the grain weight is strongly correlated with the number of endosperm cells in rice (Yang et al. 2002). Because GFR and the starch accumulation rate (SAR) per cell in IR36 would be underestimated if the differences in GFR and SAR between IR36 and T65 were compared using the absolute values of grain weight and starch content, the relative values for each final weight were used to compare GFR and SAR between the two cultivars (Fig. 1b, c, e and f). As shown in Fig. 1c and f, GFR and SAR in IR36 were significantly higher than in T65 from 8 to 14 days after flowering (DAF). Thus, IR36 seemed to be able to synthesize endosperm starch faster than T65, resulting in early maturation of IR36.

**Fig. 2:**
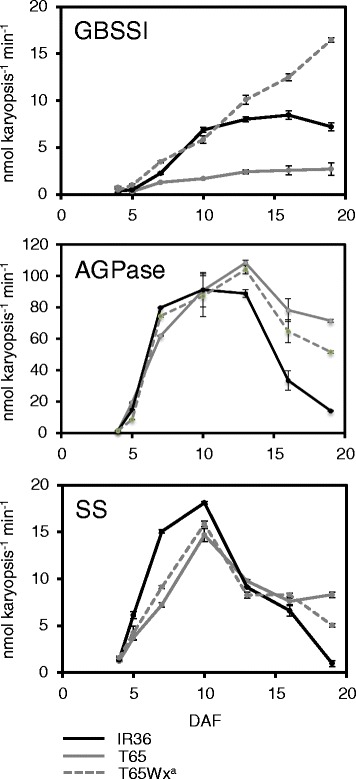
Activity of ADP-glucose pyrophosphorylase (AGPase), granule-bound starch synthase I (GBSSI) and starch synthases (SSs) in endosperm of rice cultivars IR36, T65 and T65Wx^a^ over time during ripening. Error bars indicate the SE for biological triplicates. DAF, days after flowering

**Fig. 3:**
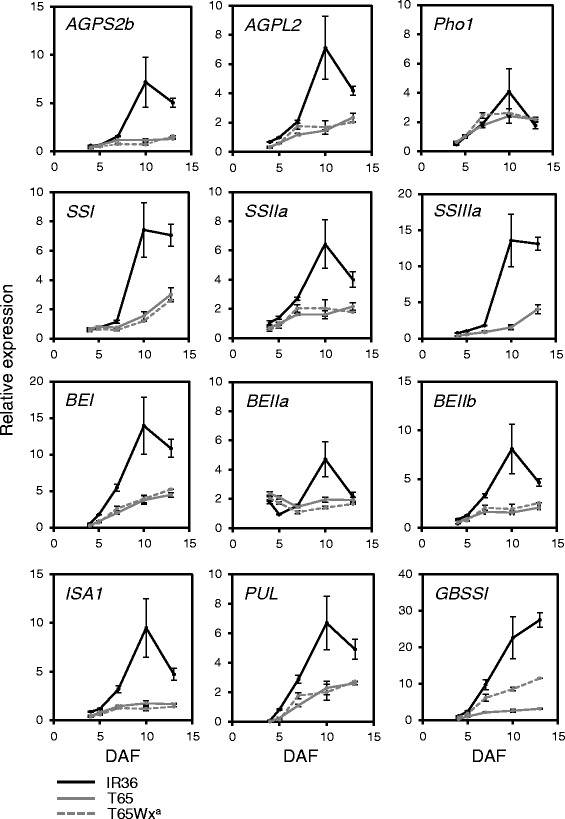
Relative transcript levels of genes involved in starch synthesis in endosperm over time during ripening period among rice cultivars IR36, T65 and T65Wx^a^. The relative ratios were calculated using the geometric mean of the four internal standard genes *actin1, eEF-1a, eIF-4a and α-tubulin.* Error bars indicate the SE for biological triplicates. DAF, days after flowering

To elucidate the factors responsible for the difference in SAR between IR36 and T65, we first investigated the relationship between SAR and the activity of the amylose-synthesizing enzyme GBSSI. Because IR36 has the wild-type allele *Wx* ^*a*^ and T65 has the mutant allele *Wx* ^*b*^ and thus lower amylose synthesis, the difference in SAR could be due to this difference in amylose synthetic activity. Indeed, *Wx* ^*a*^ gene expression level and GBSSI activity in IR36 was about 4× and 10× higher, respectively, than in T65 at 10 DAF (Figs. 2, 3). Thus, the amylose accumulation rate (AsAR) was higher in IR36 than in T65 (Fig. 1h). To determine whether this difference in AsAR affected SAR, the SAR in a T65 near-isogenic line carrying *Wx* ^*a*^ (T65Wx^a^) was compared with that of T65. As in IR36, T65Wx^a^ had high *Wx* ^*a*^ expression, GBSSI activity and AsAR (Figs. 1h, 2, 3); however, the SAR in T65Wx^a^ was similar to T65 and thus lower than in IR36 (Fig. 1f). These results indicated that elevation of amylose synthetic ability alone is not sufficient for the increase in SAR in rice endosperm, probably because enzymes for the synthesis of amylose and for amylopectin compete for the same substrate, ADP-glucose. Thus, the high SAR of IR36 appears to be due to either the high activity for the synthesis of both amylose and amylopectin or only for amylopectin.

We next compared the total activities of AGPases that synthesize ADP-glucose, a substrate for the synthesis of both amylose and amylopectin and of the SSs that are in involved in amylopectin synthesis. For the total AGPase activity, a slight but significant difference was found between IR36 and T65 at 7 DAF (*P* < 0.001; Fig. 2); however, the pattern of AGPase activity over time was almost similar between IR36 and T65 until IR36 had matured (Fig. 2). On the other hand, the total activity of SSs from 5 to 10 DAF was always higher in IR36 than in T65, and at the peak level at 7 DAF was about 2× higher than in T65 (*P* < 0.001) (Fig. 2). This result was highly consistent with the differences in SAR observed between IR36 and T65 and strongly suggested that a high synthesis of amylopectin was one factor leading to the high SAR in IR36.

To analyze the factors contributing to the difference in amylopectin synthesis between IR36 and T65, we analyzed the expression patterns of the genes related to amylopectin synthesis from 4 to 13 DAF. The expression of SS, BE and DBE genes sharply increased from 7 to 10 DAF in IR36, as opposed to a rather gradual increase in T65 (Fig. 3), so that the expression of all amylopectin synthetic genes was several-fold higher in IR36 by 10 DAF (*P* < 0.05 for all amylopectin synthetic genes; Fig. 3). Although the expression of *AGPS2b* and *AGPL2* genes was also remarkably upregulated in IR36 compared with T65 (Fig. 3), such proportional differences in the total AGPase activity between IR36 and T65 were not found (Fig. 2). The expression of *Pho1*, which encodes a plastidial phosphorylase, involved in the synthesis of glucan primers, was similar between IR36 and T65 (Fig. 3). Taken together, these results suggested that enzymes in the pathway for amylopectin synthesis were highly active in IR36, leading to the high SAR.

### Sugar-dependent and -Independent Regulation of Starch-synthetic Gene Expression in Endosperm

**Fig. 4:**
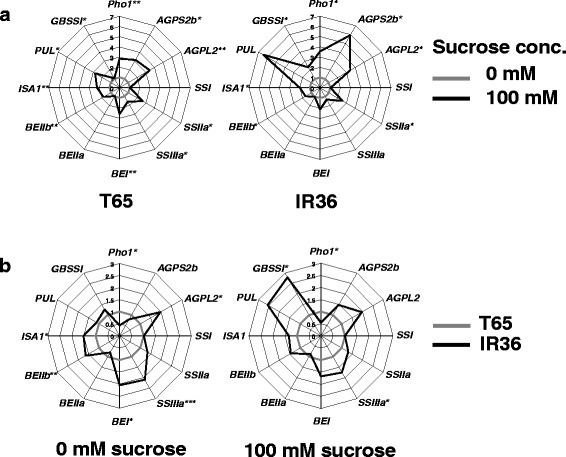
Expression profiles for starch-synthetic genes in endosperm in cultured panicles at 5 DAF of rice *indica* cultivar IR36 and *japonica* cultivar T65. **a** Effect of 0 and 100 mM sucrose on gene expression in each cultivar. Expression is given relative to the value at 0 mM sucrose. **b** Differences in gene expression between IR36 and T65 exposed to 0 or 100 mM sucrose. Significant differences were determined using biological triplicates and Student’s *t*-test (*P ≤ 0.05, **P ≤ 0.01, ***P ≤ 0.001)

**Fig. 5:**
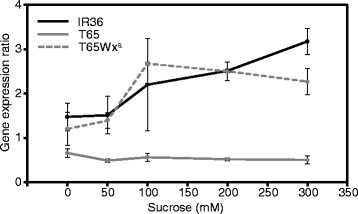
Effects of different sucrose concentrations on expression of *Wx* ^*a*^ and *Wx* ^*b*^ genes. Error bars indicate the SE for biological triplicates. DAF, days after flowering

To determine the regulatory pathway(s) that contribute to the differential regulation of the SSGs between IR36 and T65, we investigated the response patterns of each SSG to sucrose in panicles that had been harvested at 3 DAF, then cultured in water at 25 °C for 24 h. After panicle transfer to 0 mM or 100 mM sucrose and incubated at 25 °C for 24 h, the expression of all SSGs except *SSI* and *BEIIa* in both cultivars had increased in response to 100 mM sucrose (Fig. 4a). Only the *GBSSI* in IR36 (*Wx* ^*a*^) was responsive to sucrose, not that in T65 (*Wx* ^*b*^) (Fig. 4a). Thus, most SSGs in endosperm were regulated in a sucrose-dependent manner. To confirm whether *Wx* ^*b*^ in T65 had completely lost responsiveness to sucrose, we investigated the change in expression of *Wx* ^*b*^ and *Wx* ^*a*^ as sucrose levels varied from 0 to 300 mM. The expression of the *Wx* ^*a*^ genes in both IR36 and T65Wx^a^ increased with increasing sucrose concentration, while the expression of *Wx* ^*b*^ in T65 was not upregulated even at 300 mM sucrose, indicating that *Wx* ^*b*^ was mostly insensitive to sucrose (Fig. 5). On the other hand, when the gene expression data were compared between IR36 and T65 at the 0 mM sucrose level, the expression of *AGPL2*, *SSIIIa*, *BEI*, *BEIIb* and *ISA1* were 1.5 to 2× higher in IR36 than in T65, while *Pho1* expression was 2× lower in IR36 than in T65 (Fig. 4b). These data suggested that some SSGs were also regulated independently of sucrose signals. At 100 mM sucrose, the comparative patterns of the SSGs, except *GBSSI*, *AGPS2b* and *PUL*, between IR36 and T65 were almost similar to those at 0 mM sucrose (Fig. 4b), indicating that *GBSSI*, *AGPS2* and *PUL* in IR36 were more responsive to sucrose than those in T65; the response patterns of the rest of the SSGs were well conserved between IR36 and T65.

Thus, the expression of some SSGs in rice was regulated in at least two ways, namely, a sucrose-dependent and a sucrose-independent manner. In both regulatory modes, distinct differences were found between IR36 and T65. The expression level of *SSI* and *BEIIa* in the cultured panicles was almost similar between IR36 and T65, regardless of the sucrose concentration (Fig. 4). However, because the expression of both genes was considerably upregulated in IR36 than T65 at 10 and/or 14 DAF under typical growth conditions (Fig. 3), an unknown pathway(s) might be still involved in regulating the expression of the SSGs in rice endosperm.

**Fig. 6:**
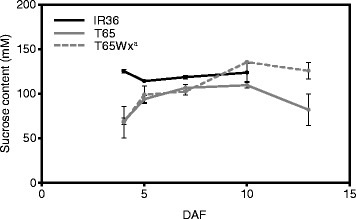
Sucrose content in crude extracts of developing karyopses of IR36, T65 and T65Wx^a^ during ripening. Error bars indicate the SE for biological triplicates. DAF, days after flowering

In the sucrose-dependent regulation of SSGs, those expression levels appeared to be determined by the sucrose concentration in the endosperm cells. When we assayed the sucrose concentration in a crude extract of developing karyopses during ripening, the sucrose concentration in IR36 was mostly constant at about 120 mM, while the sucrose level in T65 was lower (70–90 mM) from 4 to 5 DAF and only reached 100 mM by 7 DAF (Fig. 6). These results suggested that the differences of the SSG expression between IR36 and T65 might also be indirectly caused through sucrose-dependent regulation.

## Discussion

In our analyses of the difference in SAR between *indica* and *japonica* rice cultivars from the viewpoint of sink potentials, the higher starch accumulation in IR36 mainly depended on greater amylopectin synthesis; most genes involved in amylopectin synthesis were highly upregulated in IR36. The SSGs were regulated in either a sucrose-dependent or -independent manner, or both, and other regulation pathways might also be involved in the expression of SSGs such as *SSI* and *BEIIa*. In IR36, all these regulatory systems for amylopectin synthesis were more active than in T65.

Although some SSGs were regulated by multiple systems, which regulatory systems were most crucial for achieving the higher expression of those SSGs in IR36 was unclear. In panicle culture without sucrose, *AGPL2*, *SSIIIa*, *BEI*, *BEIIb* and *ISA1* genes of IR36 were more upregulated than in T65 (Fig. 4b). This sucrose-independent regulation appeared to define the basal level of the SSG expression; these SSG levels increased further in a sucrose-dependent manner (Fig. 4a). Although the sucrose-response patterns of most SSGs for amylopectin synthesis were similar between IR36 and T65 (Fig. 4), this sucrose-dependent regulation could also indirectly contribute to the high SSG expression in IR36. Actually, the sucrose concentration in crude extracts from developing seeds of IR36 was higher than in those of T65 in the early to middle phase of ripening (Fig. 6), so expression of the sucrose-responsive SSGs was expected to be higher in IR36 than in T65 during that period.

In this study, we did not obtain any information on the regulation of the *SSI* and *BEIIa* genes. For these genes, unknown signals might be involved in the regulation of their expression. For instance, Akihiro et al. (2005) reported that the expression of the plastidial *AGPL3* gene was significantly enhanced by exogenous application of ABA to rice suspension culture cells in the presence of sucrose. Interestingly, only ABA treatment decreased the expression of *OsAPL3* (Akihiro et al. 2005). These facts suggest that not only ABA but also the interaction of ABA with a sucrose signal are important to activate expression of *APGL3*. However, neither *SSI* nor *BEIIa* were upregulated by 10–100 mM ABA plus 100 mM sucrose in our preliminary results (unpublished data). Studies on the involvement of other hormone signals and/or their synergistic effects with sucrose signals in starch synthesis are needed to better understand the regulation of SSGs in rice endosperm.

Sugars function as signal molecules in plant development, growth and responses to environmental stresses (Rolland et al. 2006; Eveland and Jackson 2012; Lastdrager et al. 2014). Sugar signals, as we have shown here, apparently also play important roles in defining source–sink relationships in rice. The sink potential of rice endosperm is partly determined by the amount of translocated sugar supplied from the source organs; sink strength is always coordinated with the strength of the source such as the productivity of photosynthesis in leaves and/or the efficiency of sugar translocation through phloem. It is noteworthy that the sucrose-dependent regulation was not uniform among genes for amylose and amylopectin syntheses, suggesting that any fluctuation in sucrose translocation may affect amylopectin structure and/or the ratio of amylopectin to amylose.

We found a distinct difference in sucrose responsiveness between the *Wx* ^*a*^ and *Wx* ^*b*^ alleles (Figs. 4 and 5). *Wx* ^*a*^ was highly responsive to sucrose while the sucrose responsiveness in *Wx* ^*b*^ appeared to be almost lost. So far, the difference in the expression levels between *Wx* ^*a*^ and *Wx* ^*b*^ has mainly been explained by a decline in splicing efficiency caused by the base substitution at the splicing site of intron 1 of *Wx* ^*b*^ (Bligh et al. 1998; Cai et al. 1998; Isshiki et al. 1998; Hirano et al. 1998). However, other factors such as a difference in sucrose responsiveness could also be involved in the differential regulation in the *Wx* gene. Because the sucrose-response pattern of *Wx* ^*a*^ in T65Wx^a^ was similar to that in IR36, the difference in the sucrose response between *Wx* ^*a*^ and *Wx* ^*b*^ might be due to the differences in the *cis*-acting regulatory sequences.

Because T65 possessed the *alk* allele at the *Alk* locus encoding SSIIa (data not shown), SSIIa enzyme activity in T65 should be nearly lost after the substitution a few amino acids (Nakamura et al. 2005). Therefore, the lower SSs activity in T65 compared with IR36 might be due not only to a reduction in the level of SSGs but also to a decline in SSIIa activity. To elucidate to what extent each enzyme encoded by the SSGs, including SSIIa, is rate-limiting for starch synthesis in the endosperm, we need to develop and analyze a series of IR36 mutants for each SSG.

During ripening, the AGPase activity between IR36 and T65 differed little although the expression of both *AGPS2b* and *AGPL2* genes was much higher in IR36 than in T65. In rice endosperm, AGPase is positively regulated by 3-phosphoglyceric acid (3-PGA) and negatively by inorganic phosphate (Pi) (Sikka et al. 2001; Sakulsingharoj et al. 2004; Tuncel et al. 2014). Although it is still uncertain how 3-PGA and Pi can signal the availability of carbon and energy for starch synthesis in the endosperm, the cytosolic AGPase activity in rice endosperm might be maintained at a certain level due to such allosteric regulation although the expression levels of *AGPS2b* and *AGPL2* fluctuated. We also showed that the cytosolic *AGPS2b* and *AGPL2* genes were highly upregulated at 100 mM sucrose in endosperms of both IR36 and T65. However, such high responsiveness to sucrose in those genes was not observed in suspension culture cells of *japonica* cultivar Nipponbare (Akihiro et al. 2005). These differences suggest that the expression of the *AGPS2b* and *AGPL2* genes might be under tissue-specific regulation or vary among *japonica* cultivars.

The SAR did not significantly increase in T65Wx^a^ with high amylose synthesis. When only amylose synthesis activity increased, why was SAR not elevated? Can the activation of both amylose and amylopectin synthesis increase SAR? According to Martin and Smith (1995), amylopectin synthesis begins before amylose synthesis and that amylose is later synthesized within developing starch granules because GBSSI is confined inside the starch granule by its own binding to the granule. Therefore, inside developing granules, enzymes for the amylose and amylopectin syntheses could equally compete for the same substrate, but on the surfaces of developing granules, amylopectin synthesis might proceed preferentially and not compete with amylose synthesis. For such reasons, activation of only amylose synthesis is not considered to be responsible for the higher SAR in IR36. For high SAR in rice endosperm, higher amylopectin synthesis activity seems to be essential.

Although the expression level of SSI in IR36 was much higher than in T65 during ripening, Takemoto-Kuno et al. (2006) reported that the SSI expression in the *indica* cultivar Kasalath was lower than in the *japonica* cultivar Nipponbare. These facts suggest that the regulation of SSGs may be quite variable within and between subspecies. Because TFs of SSGs in rice, RSR1 and OsbZIP58, were previously reported and their target SSGs were characterized (Fu and Xue 2010; Wang et al. 2013), we know that RSR1 targets all SSGs, whereas only some of the SSGs are targeted by OsbZIP58. Functional variation in such master regulators of SSGs might be one factor leading to the diversification of the SSG expression pattern in rice endosperm.

## Conclusions

In this study of potential sink factors leading to the high SAR in IR36, we showed that a high level of amylopectin synthesis was crucial for the high starch synthesis in IR36. The SSG regulatory systems in the rice endosperm are rather complicated; at least three pathways are probably involved in the signaling to activate SSG expression. At the basal level, the SSGs in IR36 seemed to be more highly expressed than in T65, and IR36 expression levels increased more due to a sucrose-dependent pathway and/or pathways involved in unknown signals. Although we did not deal here with varietal differences in source strength, the sucrose concentration in the karyopsis tissues of IR36 appeared to be maintained at a higher level than in T65, especially during early to mid ripening, suggesting that the source strength of IR36 was more reinforced than T65. Thus, the high SAR in IR36 appears to be achieved by a well-coordinated balance of source supply and sink demand.

## Methods

### Plant Materials and Growth Conditions

Rice cultivar IR36 (subsp. *indica*) and T65 (subsp. *japonica*) were used in this study. The T65 near-isogenic line (NIL) carrying the *Wx* ^*a*^ gene, T65Wx^a^, was also used (Mikami et al. 1999). Seeds of T65Wx^a^ were obtained through the courtesy of Dr. Y. Sano, Graduate School of Agriculture, Hokkaido University. For measuring amylose, starch from two amylose-free lines T65wx and TR60 were used as standards for amylopectin. T65wx is a T65 NIL carrying *wx* (Mikami et al. 1999); TR60 was a F_3_ line derived from the cross between T65wx and IR36 and possesses *wx* and *Alk*. Plants were grown in the greenhouse of Hokkadio University at Sapporo from April to August. Sowing dates for IR36, T65 and T65Wx^a^ were adjusted so that spikelets from IR36, T65 and T65Wx^a^ for all experiments were flowering at the same time.

### Panicle Culture

Rice panicles were cultured by the method of Hirano and Sano (1998). Briefly, rice panicles with the stem were sampled 3 DAF, and samples were then cut at the node just beneath the panicle with a razor in water. The panicle separated from the stem was immediately transferred to a test tube including 5 ml of water and covered with a plastic bag to prevent drying. After 24 h, the panicle was transferred to another test tube with 5 ml of water or 100 mM sucrose solution and incubated for 24 h. The sucrose concentration in the culture medium was determined by previously described methods for rice (Hirano and Sano 1998; Lee et al. 2000; Kobata et al. 2001). Developing karyopses for expression analyses were then carefully excised with forceps.

### Measurement of Dry Mass, Starch, Amylose and Sucrose

Spikelets that flowered at the same time were marked with a water-based marker, and 20–30 developing karyopses per cultivar were collected at a certain interval. For measuring dry mass, samples were kept in an aluminum can and dried at 105 °C. After 12 h, they were cooled to room temperature in a dessicator, and the mass was measured. These weighed samples were then used to determine the starch content using the glucoamylase-glucose oxidase method (Thivend et al. 1965). Based on the data for dry mass and starch content, the amount of starch per karyopsis was calculated. For amylose, developing karyopses were dried at 40 °C, starch granules were extracted (Yamamoto et al. 1973), and apparent amylose content was then measured using iodine colorimetry (Juliano 1971; Yamakawa et al. 2007). A starch sample from amylose-free line T65wx was used as an amylopectin standard to measure amylose content for T65 and T65Wx^a^. The F_3_ line TR60, carrying both *wx* and *Alk*, was selected from the population derived from a cross between IR36 and T65wx and used as an amylopectin standard for IR36.

The amount of amylose per karyopsis was calculated from the starch mass per karyopsis and the amylose content. Sucrose concentration in crude extracts from developing karyopses was measured using the Sucrose Assay Kit, EnzyChrom (BioAssay Systems, Hayward, CA, USA) following the manufacturer’s instructions.

### Enzyme Activity Assay

Developing karyopses were homogenized using a mortar and pestle on ice in 4–10 volumes of a grinding solution of 50 mM HEPES-NaOH (pH 7.4), 2 mM MgCl_2_, 50 mM 2-mercaptoethanol and 12.5% (v/v) glycerol for assay of AGPase or 50 mM Tris-HCl (pH 7.4), 2 mM EDTA, 5 mM dithiothreitol, 0.4 mM phenylmethylsulfonyl fluoride and 12.5% (v/v) glycerol for the SS and GBSSI assays. The homogenates were centrifuged at 14,000 rpm at 4 °C for 15 min, and the supernatants were used as the crude enzyme extract for the AGPase and SS assays, respectively. For the GBSSI assay, the precipitated starch granules were used as the crude enzyme extract. AGPase, SS and GBSSI were assayed using the methods of Nishi et al. (2001). Three separate extracts were analyzed.

### Gene Expression Analysis by Quantitative RT-PCR

Total RNA was extracted from developing karyopses using TRIzol reagent (Invitrogen, Tokyo) and the manufacturer’s instructions, then treated with RNase-free DNase-I (Roche Diagnostics, Mannheim, Germany) to remove DNA contamination. The expression of the genes involved in starch synthesis of rice endosperm (Ohdan et al. 2005; Satoh et al. 2008) were assayed by the multiplex RT-PCR method using the GenomeLAB GeXP Start Kit (Beckman Coulter, Fullerton, CA, U. S. A.) as described previously (Kim et al. 2008). As internal standards, the *actin1, eEF-1a, eIF-4a and α-tubulin* genes were chosen according to Li et al. (2009). Expression of the starch-synthetic genes was calculated as a relative ratio to the geometric mean of the four internal standard genes (Vandesompele et al. 2002). The sequences of the primers used in this study are summarized in Additional file 1: Table S1.

## Additional file

Additional file 1: Table S1. List of primers used for expression analysis. (XLSX 31 kb)

## Abbreviations

3-PGA: 3-phosphoglyceric acid
AA: Amino acid
ABA: Abscisic acid
AGPase: ADP-glucose pyrophosphorylase
AsAR: Amylose accumulation rate
BE: Starch branching enzyme
DAF: Days after flowering
DBE: Starch debranching enzyme
GBSSI: Granule-bound starch synthase I
GFR: Grain filling rate
ISA1: Isomerase 1
NIL: Near-isogenic line
Pho1: Plastidial starch phosphorylase
Pi: Inorganic phosphate
PUL: Pullulanase
SAR: Starch accumulation rate
SS: Starch synthase
SSG: Starch-synthetic gene
T65: Taichung 65
